# Housing wealth appreciation and heterogeneous household consumption: Evidence from China

**DOI:** 10.1371/journal.pone.0289712

**Published:** 2023-10-05

**Authors:** Yingying Qi, Guohua Yu, Xiang Liu, Yuanming Ren

**Affiliations:** 1 College of Economics, Sichuan University, Chengdu, China; 2 College of Economics and Management, Southwest University, Beibei District, Chongqing, China; 3 Business School, Sichuan University, Chengdu, China; 4 Management Committee of Rongchang Campus, Southwest University, Rongchang District, Chongqing, China; University of Almeria, SPAIN

## Abstract

In this paper, we develop a DSGE model including heterogeneous households, introduce the financial friction of credit constraint mechanism, and study the impact of house price shocks on the consumption of heterogeneous household. Based on this, the CHFS data in 2011, 2013, 2015, 2017, and 2019 were used to test the marginal propensity to consume for housing wealth appreciation under different credit constraints. Results show that: Firstly, the financial accelerator mechanism plays an important role in the transmission of housing price shocks to household consumption. The looser the degree of credit constraints, the more obvious the rise in housing prices will be to the consumption expenditure of borrowing household. Secondly, the impact of housing wealth appreciation on household consumption under different credit constraints is heterogeneous. Among them, housing wealth appreciation has a significant positive impact on household consumption expenditure with multiple houses, credit cards, non-loan restrictions, while the marginal effect on the consumption expenditure of households with only one house, loan limited, and no credit cards decreases. Thirdly, for every 1% increase in the housing wealth appreciation, household consumption will increase significantly by 0.10–0.14%.

## 1. Introduction

Consumption has always been an important topic of concern to all sectors of society, because improving the level of consumption can increase individual utility and promote economic development. As the largest developing country in the world, China is a country with a large population and consumption. According to the China Statistical Yearbook, ratio of per capita consumption expenditure to per capita GDP dropped from 47.79% in 1978 to 30.41% in 2019, far below the world level of around 60%. Moreover, the year-on-year growth rate of per capita consumption expenditure has also shown a downward trend since 2013, from 9.61% to -4.00% in 2020. It can be seen that in the process of China’s economic rise and gradually shifting to high-quality development, the low consumption rate of residents and the slowdown in growth have hindered economic growth to some extent. Moreover, China’s economic growth momentum has changed, and the economic model driven by investment and exports is unsustainable. Therefore, enhancing the fundamental role of consumption in economic growth is of great significance.

With the continued prosperity of China’s economy and the gradual improvement of the financial system, household assets have shown the characteristics of scale expansion and structural diversification. In 2016, housing assets accounted for nearly 70% of total household assets in china. Therefore, housing wealth may be an important factor affecting household consumption. Over the past several decades, the relationship between housing wealth and household consumption for both developing and developed countries has been extensively researched. Scholars pointed out that the “wealth effect” of property appreciation and rising rents, as well as property income, profoundly affects household consumption, such as Case et al. (2005), Zhang et al. (2016), Zhao et al. (2020) [1–3]. Throughout the existing studies, the relevant literature can be summarized into three points:

Firstly, real estate affects household consumption through the “wealth effect” channel. Most studies have direct evidence that rising housing prices or increased property wealth promote household consumption. Cooper (2013) pointed out that rising house prices affect household consumption by alleviating household budget constraints or credit constraints [4]. Real estate affects household expenditure through credit mortgage channels rather than pure wealth effect channels. The consumption of borrowing-restricted households will significantly increase by 0.06–0.18 dollars for every dollar increase in their housing value. Aladangady (2017) believes that increasing housing wealth can promote household consumption and have a positive wealth effect [5]. Benmelech et al.(2017) found that households spend on average $3,700 more in the months before and the first year following a home purchase [6]. He and Yang (2019) investigated in detail the difference in the impact of housing price-to-income ratio on consumption from the perspective of family and real estate characteristics. The study found that a high housing price-to-income ratio has a positive correlation with household consumption. The wealth effect of households with rented houses is less affected by the weakening of the high housing price-to-income ratio [7]. Stroebel and Vavra (2019) analyzed the complex interaction between housing prices, local demand, and retail prices. Research has shown that high housing prices increase the consumption demand of local residents, thereby increasing the profit level of retailers [8]. Choi and Zhu (2022) studied the impact of housing wealth on household consumption and found that the overall impact of housing wealth on consumption is positive and significant. When housing prices rise, households do indeed increase non housing consumption by extracting net housing value [9].

Secondly, real estate affects household consumption through the “preventive savings effect” channel. Judging from the basic conclusions in the research field of precautionary savings, some scholars pointed out that rising housing prices make residents have a strong subjective feeling about the uncertainty of the future, in order to prevent this uncertainty may lead to a decline in future living standards, residents will reduce current consumption and increase their savings to smooth future consumption. The research of Li and Huang (2015) shows that the rise of house prices will increase the household savings rate, because when house prices continue to rise, residents save for house purchase and housing loan repayment, which pushes up the savings rate [10]. Peng et al. (2019) found that the appreciation of housing wealth leads to an increase in household consumption, especially for consumer goods with high expenditure elasticity, indicating that the increase in housing wealth not only promotes household consumption but also improves consumption structure. Moreover, preventive savings are the main mechanism by which China’s housing wealth appreciation affects consumption [11]. Yang and Zou (2020) found that rural residents with low net worth of real estate face greater income risks than urban residents with high net worth of real estate, leading to stronger incentives for rural residents to save. In addition, home ownership also has a consumer hedging effect, that is, when the housing price risk is high, the rent risk is correspondingly higher [12]. Family-owned housing can hedge rent risk, avoid the uncertainty of future housing consumption, and reduce the willingness to cash in real estate [13, 14]. As a result, the risk of house price and rent fluctuations in the case of high house prices will further increase, and the “preventive savings effect” and the increase in rent risk caused by the increased risk of house price fluctuations may weaken the “wealth effect” of real estate.

Thirdly, real estate affects household consumption through the “liquidity restraint effect” channel. This effect is mainly reflected in the mortgage refinancing ability of real estate value. Rising real estate value increases the value of real estate as collateral. Households with houses can obtain loans from financial institutions and promote consumption by mitigating liquidity constraints [15–21]. A study by DeFusco (2018) found that the increase in house prices significantly promoted the refinancing of households with houses. The marginal effect of household refinancing caused by a rise of 1 dollar was between 0.04 and 0.13 [22]. At the same time, the increase in households’ access to credit funds further increased expenditures. Cloyne et al. (2019) studied the impact of housing price on household borrowing by using the official mortgage data of the United Kingdom, and found that the impact of housing price rise on household borrowing is significant, and its impact on household borrowing can be explained to a large extent by the “wealth effect” generated by housing price rise [23]. In contrast, if housing prices drop significantly, the bank may also re-estimate the value of the house from the perspective of credit risk, and at the same time require the homeowner to provide more credit default protection, thereby strengthening mobility constraints for homeowners. Lustig and Nieuwerburgh (2005) pointed out that falling house prices will reduce the mortgage value of houses, expand households’ exposure to special risks, and increase the market risk of real estate mortgage financing [24]. Dynan (2012) pointed out that in a market where the housing price-to-income ratio is relatively high and the risk of housing prices is relatively high, financial institutions will be more cautious in determining the proportion of real estate mortgage loans, reducing financial support to households, and the mortgage financing capacity of household real estate wealth will be relatively weakened, its effect on alleviating liquidity constraints and increasing consumption will also be relatively weakened [25]. Garriga et al. (2019) found that a short-term decrease in mortgage interest rates and an increase in loan to value ratio may lead to significant fluctuations in housing prices, rather than relatively small changes in housing consumption and rent [26]. Mian and Sufi (2022) document evidence that the expansion of mortgage credit supply promotes speculative housing transactions and exacerbates house price fluctuations [27]. Dong et al. (2022) believe that the expansion of credit supply has increased the housing demand of optimistic homebuyers and boosted housing prices without affecting rent. Therefore, there is a positive correlation between credit supply and housing prices [28].

The relationship between housing wealth and consumption has gained substantial attention from academics and policymakers, existing studies have provided a variety of explanations for this paper to understand the relationship between housing wealth appreciation and household consumption. Rising house prices will rapidly increase the stock of housing wealth owned by households and affect household consumption expenditures. Real estate contains both physical and virtual attributes, and the rise of housing price more reflects the virtual attributes of the real estate market. In fact, the physical attributes of real estate can’t be ignored, which plays an inestimable role in driving the national economy, promoting resident consumption and promoting industrial development. Therefore, based on the physical attributes of real estate, this article constructs a DSGE model that includes heterogeneous households, introduces the financial friction of the credit mortgage restraint mechanism, and analyzes the impact of housing price shocks on the consumption of savings households and borrowing households. Based on this, using the China Household Financial Survey data in 2011, 2013, 2015, 2017, and 2019, we test the marginal propensity to consume for household housing wealth appreciation under different credit constraints. Compared with the existing research, the marginal contribution of this article lies in:

Firstly, we clearly put forward the assumption that “the impact of housing wealth appreciation on household consumption needs to be discussed under different credit constraints”. Previous studies on heterogeneous household consumption mainly discussed the differences in household consumption from the perspectives of housing property rights, property size, household income, housing quantity, and asset structure. However, the investment property of real estate will produce the “wealth effect”, which will influence the consumption behavior by “magnifying” household income. For the credit-constrained households, the loss of wealth appreciation opportunities due to the inability to obtain real estate mortgage credit may eventually inhibit their income growth and consumption. Therefore, from the perspective of expanding domestic demand, it is indeed necessary to study the impact of housing wealth appreciation on the consumption of different types of households.

Secondly, by introducing the financial friction of the credit constraint mechanism, we reveal the theoretical mechanism of the impact of house price shocks on household consumption. This article integrates credit constraint and preventive savings theories, and explores heterogeneous household consumption behaviors under different credit constraint. Theoretically, the financial accelerator mechanism plays an important role in the transmission process of housing price shock to household consumption. Borrowing households tend to obtain loans by mortgaging real estate to smooth their consumption. The looser the credit constraint is, the more obvious the housing price rise will be to the consumption expenditure of the borrower households. However, this will lead to a decline in the borrowing capacity of savings households with the same quality and quantity of assets, which will make households face certain liquidity constraints, thereby reducing consumption. The theoretical framework constructed in this article is a new attempt in the field of heterogeneous household consumption research, and it also provides a new explanation for the phenomenon of household consumption that faces differences in the degree of credit constraint.

Thirdly, this article uses large-scale micro data to not only confirm that house price appreciation will promote household consumption, but also find that housing mortgage loans further amplify the “wealth effect” of real estate. The effect of housing wealth appreciation on household consumption under different credit constraints is heterogeneous. Among them, housing wealth appreciation has a significant positive impact on the consumption expenditure of households with multiple houses, non-restricted loans, and credit cards. Under the opposite conditions, housing wealth appreciation has a significant negative impact on household consumption expenditure or a small marginal contribution rate. Econometric analysis shows that the “wealth amplification effect” produced by the housing wealth appreciation will stimulate household consumption. For every 1% of housing wealth appreciation, household consumption will significantly increase by about 0.10–0.14%. These conclusions provide a basis for the government and relevant departments to regulate the real estate market and optimize the allocation of credit resources to help household consumption.

## 2. Housing price rise and heterogeneous household consumption: A DSGE model

Life cycle theory believes that families have different income levels, consumption tendency and consumption willingness at all stages of the life cycle. Moreover, in the real economy, not the family income level of every stage can perfectly support their consumption willingness, and it is inevitable that there will be credit constraint or liquidity constraint at a certain period. By the way, ‘credit constraint’ and ‘liquidity constraint’ are similar but different. Among them, ‘credit constraint’ focuses on capital availability, and ‘liquidity constraint’ emphasizes transformation ability. At present, China’s financial system is becoming more and more perfect, but the development level of credit market still lags behind developed economies. Therefore, it is reasonable to assume that families may face credit constraints. Therefore, this paper does not pay attention to the subtle difference between the two for the time being, and calls the phenomenon of ‘financial repression’ caused by the incomplete financial market or financial system when families participate in economic activities as ‘credit constraint’. This paper draws on Kiyotaki and Moore’s (1997) method of analyzing individual assets, liabilities and leverage by setting mortgage constraint coefficients (mortgage rates) [29], and referring to Iacoviello’s (2005) modeling ideas [15], constructs a small, closed, discrete-time model that includes representative households, manufacturers, commercial banks, and the government. Households are divided into savings household and borrowing household according to whether they are subject to credit constraints. Among them, savings households have bank deposits, obtain deposit interest, and provide labor to enterprises to obtain wage income, borrowing households mainly obtain loans from banks by mortgaging real estate, and earn income via working.

### 2.1. Representative household

Firstly, assuming that the utility function of savings households is based on consumption (*C*_*s*,*t*_), property holding (*H*_*s*,*t*_), and labor (*N*_*s*,*t*_), the objective function is:

$$\underset{C_{s},N_{s},B_{s},H_{s}}{max}E_{0}\sum_{t=0}^{\infty }\beta_{s}^{t}\log C_{s,t}+j_{t}logH_{s,t}-\frac{N_{s,t}^{1+\varphi }}{1+\varphi }$$ (1)

Among them, *E*_0_ is the mathematical expectation operator, $\beta_{s}^{t}$ is the intertemporal discount factor of savings households, *j* is the housing demand shock, the subscript *s* is used to distinguish the two types of households. It needs to be explained again that because savings households lack sufficient collateral, they will have certain credit constraints. The budget constraint of savings households is:

$$C_{s,t}+B_{s,t}+(1+\tau_{t})q_{t}H_{s,t}=W_{s,t}N_{s,t}+R_{t-1}B_{s,t-1}/\pi_{t}+q_{t}H_{s,t-1}+T_{s,t}$$ (2)

Among them, *q*_*t*_ is the actual price of real estate, *τ*_*t*_ is the real estate tax rate levied by the government, *B*_*s*,*t*_ and *W*_*s*,*t*_ are the actual total savings of savings households and the actual wages obtained by working, *R*_*t*−1_ is the interest rate, *T*_*s*,*t*_ is the government transfer payment. Combining Formulas (1) and (2) to construct the Lagrangian function:

$$L_{1}=\log C_{s,t}+j_{t}\log H_{s,t}-\lambda_{s,t}[C_{s,t}+B_{s,t}+(1+\tau_{t})q_{t}H_{s,t}-W_{s,t}N_{s,t}-R_{t-1}B_{s,t-1}/\pi_{t}-q_{t}H_{s,t-1}-T_{s,t}]$$ (3)

Derivative the selected variables *C*_*s*,*t*_, *N*_*s*,*t*_, *B*_*s*,*t*_ and *H*_*s*,*t*_ separately. The FOCs is:

$$\frac{1}{C_{s,t}}-\lambda_{s,t}=0$$ (4)

$$\frac{W_{s,t}}{C_{s,t}}=N_{s,t}^{\varphi }$$ (5)

$$\frac{1}{C_{s,t}}=E_{t}(\beta_{s}R_{t}/C_{s,t+1}\pi_{t+1})$$ (6)

$$\frac{(1+\tau_{t})q_{t}}{C_{s,t}}=j_{t}\frac{1}{H_{s,t}}+\beta_{s}E_{t}\frac{q_{t+1}}{C_{s,t+1}}$$ (7)

Secondly, assuming that the utility function of the borrowing household is based on consumption (*C*_*b*,*t*_), property holding (*H*_*b*,*t*_) and labor (*N*_*b*,*t*_), the objective function is:

$$\underset{C_{b},N_{b},B_{b},H_{b}}{max}E_{0}\sum_{t=0}^{\infty }\beta_{b}^{t}\log C_{b,t}+j_{t}logH_{b,t}-\frac{N_{b,t}^{1+\varphi }}{1+\varphi }$$ (8)

Since borrowing households can apply for loans from banks with real estate as collateral, the budget constraints they face are as follows:

$$C_{b,t}+R_{t-1}D_{b,t-1}+(1+\tau_{t})q_{t}H_{b,t}=W_{b,t}N_{b,t}+q_{t}H_{b,t-1}+D_{b,t}+T_{b,t}$$ (9)

$$R_{t}D_{b,t}\leq E_{t}(\rho_{b}q_{t+1}\pi_{t+1}H_{b,t})$$ (10)

Among them, *D*_*b*,*t*_ and *W*_*b*,*t*_ are the total actual deposits and the actual wages received by the borrowing household respectively, *T*_*b*,*t*_ is the government transfer payments, *ρ*_*b*_ is the ratios of mortgage loans. Constructing the Lagrangian function by Formulas (8), (9) and (10):

$$\begin{array}{l} L_{2}=\log C_{b,t}+j_{t}\log H_{b,t}-\frac{N_{b,t}^{1+\varphi }}{1+\varphi }-\lambda_{b,t}[C_{b,t}+R_{t-1}D_{b,t-1}+(1+\tau_{t})q_{t}H_{b,t}-W_{b,t}N_{b,t}-q_{t}H_{b,t-1}-D_{b,t}-T_{b,t}]\\ -\lambda_{b,t}^{B}[R_{t}D_{b,t}-E_{t}(\rho_{b}q_{t+1}\pi_{t+1}H_{b,t})] \end{array}$$ (11)

Derivative the selected variables *C*_*b*,*t*_, *N*_*b*,*t*_, *D*_*b*,*t*_ and *H*_*b*,*t*_ separately. The FOCs is:

$$\frac{1}{C_{b,t}}-\lambda_{b,t}=0$$ (12)

$$\frac{W_{b,t}}{C_{b,t}}=N_{b,t}^{\varphi }$$ (13)

$$\frac{1}{C_{b,t}}=\lambda_{b,t}^{B}R_{t}+E_{t}(\beta_{b}R_{t}/C_{b,t+1}\pi_{t+1})$$ (14)

$$\frac{(1+\tau_{t})q_{t}}{C_{b,t}}=j_{t}\frac{1}{H_{b,t}}+E_{t}(\beta_{b}\frac{q_{t+1}}{C_{s,t+1}}+\lambda_{b,t}^{B}\rho_{b}q_{t+1}\pi_{t+1})$$ (15)

### 2.2. Enterprise and retailers

Enterprise. Assuming that the production function of the firm is in the form of Cobb-Douglas, which invests capital, labor and mortgage property for production, and mortgage real estate to the commercial bank, the specific production function can be expressed as:

$$Y_{t}=A_{t}K_{t}^{\alpha }H_{e,t}^{\theta }N_{s,t}^{\omega (1-\alpha -\theta )}N_{b,t}^{\left(1-\omega )(1-\alpha -\theta \right)}$$ (16)

Among them, *K*_*t*_ and *H*_*e*,*t*_ are capital and the number of property respectively, *N*_*s*,*t*_ and *N*_*b*,*t*_ are the amount of savings household and borrowing household labor employed by firms, *ω* and 1−*ω* are the labor input shares of savings household and borrowing household, the remaining parameters *α*, *θ*, *ω*(1−*α*−*θ*) and (1−*ω*)(1−*α*−*θ*) are respectively the capital invested in the production of the enterprise, the stock of real estate, and the output share of savings-type households and loan-type households in the production function.

Further, this paper uses the perpetual storage method including “investment adjustment cost” to describe the capital accumulation process:

$$I_{t}=K_{t}-(1-\delta )K_{t-1}+\xi_{K,t}$$ (17)

The problem of maximizing entrepreneur utility is expressed as:

$$\underset{B,I,K,H,N_{s}{,}_{Nb}}{max}\;E_{0}\sum_{t=0}^{\infty }\gamma_{}^{t}logC_{e,t}$$ (18)

Among them, *γ*^*t*^ is the discount factor of entrepreneurs, *C*_*e*,*t*_ is the consumption of entrepreneurs. The budget constraints faced by enterprises are:

$$\frac{Y_{t}}{X_{t}}+D_{e,t}=C_{e,t}+q_{t}(H_{e,t}-H_{e,t-1})+\frac{R_{t-1}}{\pi_{t}}D_{e,t-1}+w_{s,t}N_{s,t}+w_{b,t}N_{b,t}+I_{t}+\xi_{K,t}+\xi_{e,t}$$ (19)

$$R_{t}D_{e,t}\leq \kappa_{e}q_{t+1}H_{e,t}\pi_{t+1}$$ (20)

Among them, *D*_*e*,*t*_ is the amount of loan that the business actually receives from a commercial bank, $X_{t}=P_{t}/P_{t}^{w}$ is the price markup, $P_{t}^{w}$ is the wholesale price of the products produced by the enterprise, and the retailer sells the products to the household at the final price (*P*_*t*_) after the integration of the enterprise’s products. Let *Q*_*t*_ is the nominal price of real estate, then the actual price and real wage of real estate are *q*_*t*_ = *Q*_*t*_/*P*_*t*_ and *w*_*t*_ = *W*_*t*_/*P*_*t*_ respectively. Further express the investment adjustment cost as:

$$\xi_{K,t}=\frac{\varphi_{K}}{2}{\left(\frac{I_{t}}{K_{t-1}}-\delta \right)}^{2}\times K_{t-1}$$ (21)

$$\xi_{e,t}=\frac{\varphi_{H}}{2\delta }{\left(\frac{H_{{}_{e,t}}-H_{{}_{e,t-1}}}{H_{{}_{e,t}}}\right)}^{2}\times H_{{}_{e,t-1}}$$ (22)

Then, let *λ*_*e*,*t*_, $\lambda_{e,t}^{B}$, $\lambda_{e,t}^{K}$ are respectively the Lagrangian multiplier of corporate budget constraint, the Lagrangian multiplier of mortgage constraint and the shadow price of investment. Constructing Lagrangian function by Formulas (17), (18), (19) and (20):

$$\begin{array}{l} L_{3}=E_{t}\sum_{t=0}^{\infty }\gamma_{}^{t}\{logC_{e,t}+\lambda_{e,t}[\frac{Y_{t}}{X_{t}}+D_{e,t}-C_{e,t}-q_{t}(H_{e,t}-H_{e,t-1})-\frac{R_{t-1}}{\pi_{t}}D_{e,t-1}-w_{s,t}N_{s,t}-w_{b,t}N_{b,t}-I_{t}-\xi_{K,t}-\xi_{e,t}]\\ -\lambda_{e,t}^{B}(R_{t}D_{e,t}-\kappa_{e}q_{t+1}H_{e,t}\pi_{t+1})-\lambda_{e,t}^{I}[K_{t}-(1-\delta )K_{t-1}-I_{t}]\} \end{array}$$ (23)

Derivative the variables *C*_*e*,*t*_, *D*_*e*,*t*_, *I*_*t*_, *K*_*t*_, *H*_*e*,*t*_, *N*_*s*,*t*_ and *N*_*B*,*t*_. separately. The FOCs is:

$$\frac{1}{C_{e,t}}-\lambda_{e,t}=0$$ (24)

$$\frac{1}{C_{e,t}}=E_{t}\gamma (\frac{R_{t}}{C_{e,t+1}\pi_{t+1}})+\lambda_{e,t}^{B}R_{t}$$ (25)

$$\lambda_{e,t}^{I}=\frac{1}{C_{e,t+1}}[1+\frac{\varphi }{\delta }(\frac{I_{t}}{K_{t+1}}-\delta )]$$ (26)

$$\lambda_{e,t}^{I}=E_{t}\gamma \frac{1}{C_{e,t+1}}[\frac{\varphi }{\delta }(\frac{I_{t+1}}{K_{t}}-\delta )\times \frac{I_{t+1}}{K_{t}}-\frac{\varphi }{2\delta }{\left(\frac{I_{t+1}}{K_{t}}-\delta \right)}^{2}]+E_{t}\gamma [\frac{\mu Y_{t+1}}{C_{e,t+1}X_{t+1}K_{t}}+\lambda_{e,t}^{I}(1-\delta )]$$ (27)

$$\frac{1}{C_{e,t+1}}q_{t}=E_{t}[\gamma \frac{1}{C_{e,t+1}}(\theta \frac{Y_{t+1}}{X_{t+1}H_{e,t}}+q_{t+1})+\lambda_{e,t}^{B}\kappa_{e}q_{t+1}\pi_{t+1}]$$ (28)

$$w_{s,t}=\frac{\omega (1-\alpha -\theta )Y_{t}}{X_{t}N_{s,t}}$$ (29)

$$w_{b,t}=\frac{(1-\omega )(1-\alpha -\theta )Y_{t}}{X_{t}N_{b,t}}$$ (30)

Retailer. In order to introduce price stickiness, the retailer is set to package and aggregate the products produced by the company according to the standard DSGE model, and sell it to the household at the final price *P*_*t*_. Pricing obeys the Phillips curve with logarithmic linearization:

$$\pi_{t}=\beta_{s}E_{t}\pi_{t+1}-\frac{\left(1-\eta )(1-\beta_{s}\eta \right)}{\eta }X_{t}$$ (31)

Among them, *η* represents the probability that the sales price changes in each period.

### 2.3. Commercial bank

In order to study the impact of household house price fluctuations on household consumption under different credit constraints, this paper introduces the financial friction of the credit constraint mechanism. We assume that a commercial bank requires households or businesses to provide a certain percentage of asset collateral for each loan. The purpose is to screen the loan risk by controlling the ratio of the credit line to the mortgage capital. Commercial banks set a credit mortgage constraint coefficient for borrowing households and enterprises, which is expressed as:

$$\mu_{b}=\frac{D_{b,t}}{E_{t}(q_{t+1}H_{b,t}\pi_{t+1})}$$ (32)

$$\mu_{e}=\frac{D_{e,t}}{E_{t}(q_{t+1}H_{e,t}\pi_{t+1})}$$ (33)

Among them, *μ*_*b*_ and *μ*_*e*_ are credit mortgage constraints coefficient. If the credit mortgage constraints coefficient is larger, it means that the commercial bank faces less risk of borrowing, and the borrowing household or enterprise can get more loans with a certain amount of mortgage capital. On the contrary, it means that the greater the risk of borrowing, the more difficult it is for households or enterprises to obtain mortgage loans.

### 2.4. Government sector

For the convenience of analysis, this article only considers the collection of real estate taxes on the household sector. The government levies real estate taxes on two types of households as fiscal revenue, and balances revenue and expenditures through transfer payments *T*_*t*_ and purchasing government expenditure *G*_*t*_. Government expenditure will not add value to households. Utility will not produce positive externalities to the company. The government budget can be expressed as:

$$G_{t}+T_{t}=\tau_{t}q_{t}H_{s,t}+\tau_{t}q_{t}H_{b,t},T_{t}=T_{s,t}+T_{b,t}$$ (34)

Among them, the real estate tax policy obeys the AR (1) process, and its logarithmic linearization form is:

$${\hat{\tau }}_{t}=\kappa_{t}{\hat{\tau }}_{t-1}+\varepsilon_{t}^{\tau }$$ (35)

### 2.5. Total resource constraints and clearance conditions

In economic operations, commercial bank credit comes from deposits of savings households, that is, commercial banks provide savings households’ deposits to borrowing households and enterprises for investment and consumption, and normalize the total amount of real estate to 1. The resource constraints of the entity can be expressed as:

$$H_{s,t}+H_{b,t}=H_{e,t}$$ (36)

$$B_{s,t}+D_{b,t}+D_{e,t}=0$$ (37)

$$Y_{t}=C_{s,t}+C_{b,t}+C_{e,t}+I_{t}+G_{t}$$ (38)

### 2.6. Numerical simulation

Parameter calibration. This article mainly studies the impact of housing wealth appreciation on household consumption under different credit constraints. In order to achieve the research goals, it is particularly important to assign values to the parameters of the model. To this end, this article will take two methods to assign values to the parameters:

Firstly, we use the settings of existing authoritative literatures to assign parameters. In the first place, set the price stickiness coefficient to 0.75, thereby ensuring that the price can be adjusted throughout the year (four quarters). Secondly, the discount factors for savings and borrowing households were set to 0.99 and 0.98, respectively, while the discount factors of entrepreneurs were set as 0.975 based on the research of Gao et al. (2018) [30]. Furthermore, regarding the capital depreciation rate, the quarterly value is 0.025, and the annual value is 0.10. At the same time, this paper estimates the proportion of fiscal expenditure to GDP in the past ten years, and the ratio is generally between 0.23 and 0.26. Therefore, the proportion of fiscal expenditure to GDP in the steady state is calibrated to 0.25. Finally, referring to Iacoviello (2005) [15], the steady-state value of housing preference shocks is calibrated to 0.124, and the standard deviation of all shocks is set to 0.10.

Secondly, Bayesian estimation is used to calibrate structural parameters and the persistence coefficient of exogenous shocks. This paper estimates the relevant parameters using four variables: China’s GDP from the first quarter of 2008 to the fourth quarter of 2019, total retail sales of social consumer goods, total investment in fixed assets, and loan balances of financial institutions. In the estimation process, the seasonal data is adjusted using the CensusX13 method, and then HP filtering is performed on the data to separate the trend item and the disturbance item, and finally the disturbance item is used as the actual data observation value for Bayesian estimation of the observation value. Among them, the estimated value of capital output elasticity is 0.5062, which is similar to the value of capital output elasticity estimated in most literatures to be between 0.4 and 0.6, the estimated value of labor share of savings-type households is 0.6263, the estimated value of the investment adjustment cost coefficient is 2.4829, the estimated value of the reciprocal of the labor supply elasticity of the household sector is 0.9620, which is similar to the estimated value 1.03 of Tan and Wang (2011) [31]. **Table 1** summarizes the values of calibration parameters:

**Table 1:** Values of the calibration parameters.

Parameter	Describe	Value	Parameter	Describe	Value
*β*_*s*_	Savings household discount factor	0.99	$\bar{G}/\bar{Y}$	Proportion of fiscal expenditure at equilibrium	0.25
*β*_*b*_	Borrowing household discount factor	0.98	*ω*(1−*α*−*θ*)	Savings household labor share	0.6263
*γ*	Entrepreneur discount factor	0.975	*j*	Real Estate Preference Coefficient	0.124
*δ*	Capital depreciation rate	0.10	*ρ*_*j*_	Random shock coefficient of housing demand	0.7937
*α*	Capital output elasticity	0.5062	*ρ*_*q*_	House price random shock coefficient	0.6783
*θ*	Property output elasticity	0.0499	*ρ*_*A*_	Technical Random Impact Coefficient	0.9524
*η*	Price stickiness coefficient	0.75	*ρ*_*K*_	Investment random shock coefficient	0.5368
*φ*	Reciprocal of labor supply elasticity	0.9620	*ρ*_*R*_	Monetary Policy Random Shock Coefficient	0.8902
*φ*_*K*_	Investment adjustment cost factor	2.4829	*ρ*_*τ*_	Property tax rate random shock coefficient	0.8056

**Fig 1:**
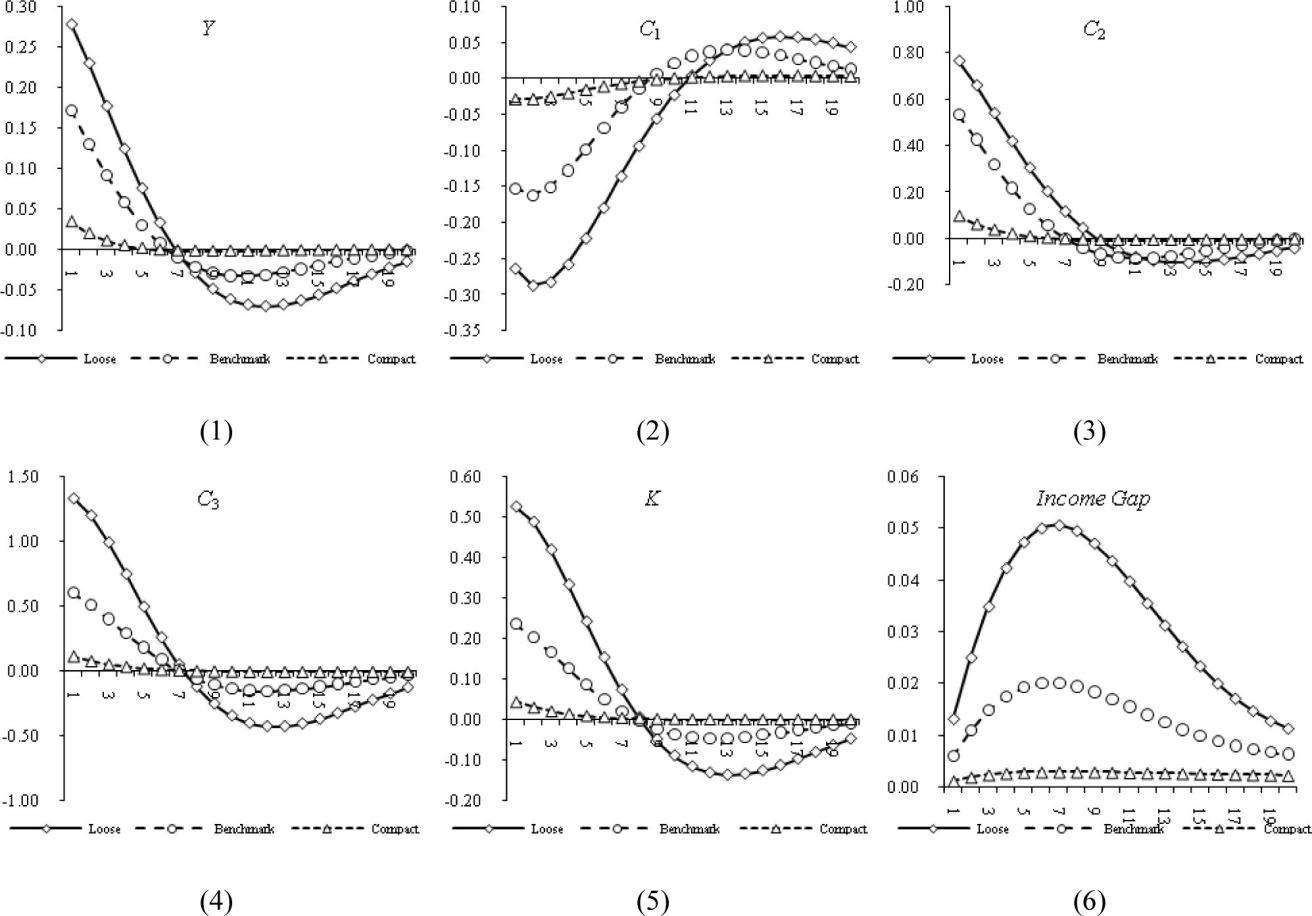
Impulse response under housing price shock.

The degree of credit constraints is closely related to the completeness of the financial market. Generally speaking, in a financial market with less financial friction and more complete information, the looser credit constraints will be. Without loss of generality, this article sets up three different credit constraint environments in the model, specifically: When *μ*_*b*_ = 0.1, *μ*_*e*_ = 0.1 indicates a financially repressive credit constraint environment, when *μ*_*b*_ = 0.7, *μ*_*e*_ = 0.8, it is consistent with the actual situation of China’s credit market, that is, the mortgage rate of households is 70%, and the mortgage of enterprises is 80%, which indicates a credit market environment with moderate mortgage constraints, and is set as the benchmark credit constrained environment, when *μ*_*b*_ = 0.85, *μ*_*e*_ = 0.9, it means that the mortgage constraint is relatively loose, that is, the credit constraint environment of financial acceleration. The pulse response under different parameter values is shown in **Fig 1**.

**Fig 2:**
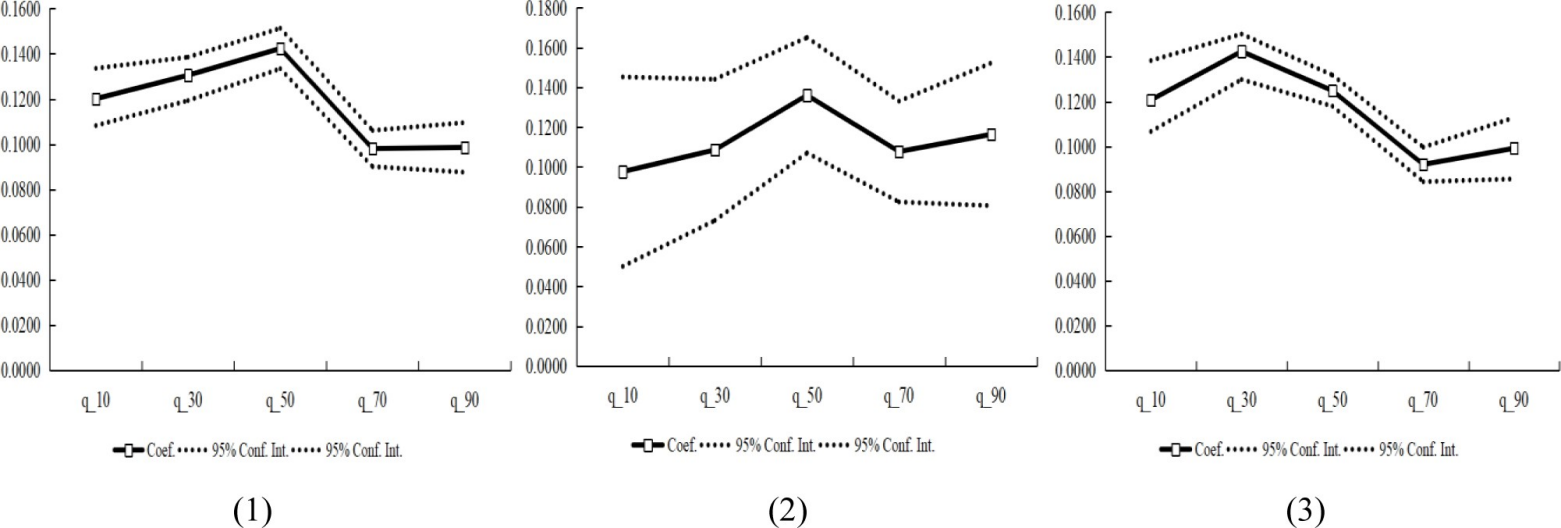
Evolution trend of marginal effect of housing wealth appreciation on household consumption.

Firstly, the sub-figure (2) in **Fig 1** plots the changes in the consumption of savings households under the impact of housing prices. In addition to analyzing the impact of house price shock on household consumption, this paper also focuses on the impact of other variables by technology shock, investment shock and monetary policy shock. In order to save space, this paper takes the impulse response of other shocks in the model as a backup cable. The results show that under different credit constraint environments, housing price shocks have a negative impact on consumption, and the looser the credit constraint environment, the greater the negative impact of housing price shocks on consumption fluctuations, which is consistent with theoretical expectations. So why does rising house prices curb savings household consumption? Theoretically, savings households cannot obtain credit funds through real estate mortgages. When the central bank adopts loose monetary policy, it means that the ability of savings households to borrow assets has declined, causing households to face a certain amount of liquidity constraints. Households need to rely on the sale of other assets for liquidity, which reduces household asset returns and thereby reduces household consumption expenditures. At the same time, most of the credit funds of commercial banks are occupied by borrowing households. Savings households cannot purchase real estate due to insufficient funds. This has also exacerbated the property income inequality between the two types of households, making savings households increase their savings and reduce consumption. At the same time, this key theoretical result has also been demonstrated by empirical studies later.

Secondly, the sub-figure (3) in **Fig 1** plots the changes in the consumption of borrowing households under the impact of housing prices. The results show that under different credit constraint environments, housing price shocks have a positive impact on consumption, and the looser the credit constraint environment, the greater the impact of housing price shocks on the positive fluctuations in consumption, which is consistent with expectations, consistent with the prediction of the model of Iacoviello and Neri (2010) [32]. The reason is that, first, from the household level, borrowing households tend to obtain loans through mortgaged real estate to smooth their consumption. The looser the degree of credit constraints, the more obvious the rise in housing prices under the financial accelerator mechanism will affect the consumption expenditure of borrowing households. On the contrary, it will inhibit the consumption expenditure of savings households, which has been demonstrated in **Fig 1**. Second, from a corporate perspective, rising housing prices have pushed up corporate net assets and relaxed corporate credit constraints. More importantly, the existence of a financial accelerator mechanism enables companies to obtain more loans for production through real estate mortgage loans. Since the production function incorporates capital and labor as complementary factors of production at the same time, the increase in capital input by enterprises will inevitably increase labor demand. Therefore, by attracting more family labor to increase the marginal output of factors, it will promote family consumption in the process of increasing family wages. Moreover, this can be confirmed from the sub-figure (6) in **Fig 1**. Under the impact of housing prices, the income gap between borrowing households and savings households shows an inverted “U-shaped” evolution trend of "expanding before shrinking". And the looser the credit constraint environment, the greater the impact of housing price shocks on the positive fluctuation of the income gap between the two types of households. It can be seen that analyzing the consumption behavior of Chinese heterogeneous households from the perspective of income inequality has a certain explanatory power.

In addition, this article also focuses on the impact of housing price shocks on other important economic variables. In particular, when the environmental parameters reflecting credit constraints (*μ*_*b*_ = 0.1 and *μ*_*e*_ = 0.1, *μ*_*b*_ = 0.7 and *μ*_*e*_ = 0.8, *μ*_*b*_ = 0.85 and *μ*_*e*_ = 0.9) change, will the impact of housing price shocks on total output, entrepreneurial consumption, investment, etc. change? Based on this idea, **Fig 1** plots the effects of house price fluctuations on total output (sub-figure 1), entrepreneurial consumption (sub-figure 4), and investment (sub-figure 5) under different credit constraints. Specifically, the impact of housing prices will increase total economic output, stimulate entrepreneurial consumption, and promote social investment, which is not only in line with theoretical expectations, but also in line with actual economic performance.

## 3. Testing strategies for heterogeneous consumer behavior differences

### 3.1. Measurement model

In order to further verify the theoretical mechanism mentioned above, this paper designs a benchmark econometric model for the impact of housing wealth appreciation on household consumption:

$$consumption_{i}=c_{1}+a_{1}house_{i}+\beta_{2}controls_{i}+\varepsilon_{i}$$ (39)

In the formula, the dependent variable is household consumption (*consumption*), the independent variable is housing wealth appreciation (*house*), *controls* is the vector of control variables, which are income (*income*), fixed assets (*asset*), cash holdings (*cash*), financial assets (*finance*), commercial insurance (*security*), borrowing scale (*debt*), human capital (*human*), gender of the head of the household (*gender*), number of households (*scale*) and financial information (*information*), *c*_1_ is the intercept item, *a*_1_ is the marginal coefficient of house price appreciation that affects consumption, *β*_2_ is the regression coefficient vector of the control variable, *ε* is residual item, *i* is the *i* household.

### 3.2. Variables

The dependent variable: Household consumption (*consumption*), which is the household consumption expenditure. In order to avoid endogeneity, household consumption does not include expenditure on housing related purchase, decoration, maintenance or expansion. It mainly covers food expenses, gas expenses (including water, electricity, fuel expenses, heating expenses, etc.), daily cosmetics, housekeeping services, transportation expenses (including self-driving oil expenses, parking expenses, repair expenses, etc.), communication expenses (including Telephone, mobile phone and other communication fees, cable television fees, etc.), cultural and entertainment expenditures (including books, magazines, movie tickets, etc.), clothing expenditures, education and training expenditures, brand-name luggage (luxury goods), education and training, travel and family visits, overseas consumption and medical care.

The independent variable: Housing wealth appreciation (*house*), we use the household housing market value minus the historical cost to measure the housing wealth appreciation. Among them, the current value of the house comes from the questionnaire stating “how much is this house worth at present”, while the historical cost of the house mainly comes from the questionnaire stating “how much did your family spend (including various taxes and fees) when you first obtained this house”. In terms of the details of data processing, this paper eliminates the samples with negative housing wealth appreciation, and divides the samples into three categories: first set of housing wealth appreciation, second set of housing wealth appreciation, and total housing wealth appreciation based on the amount of family home ownership. Among them, the appreciation of the first house refers to the situation that the nominal increment of the market value of the household’s first house minus the historical cost is greater than zero. The appreciation of second set of housing refers to the situation that the nominal increment of the market value of the household’s second set of housing minus the historical cost is greater than zero. The total appreciation of real estate refers to the situation where the sum of the nominal increment of the market value of the first house minus the historical cost and the nominal increment of the market value of the second house minus the historical cost is greater than zero.

The control variable: The selection of control variable indicators in this paper mainly refers to the principles of classic economics and existing research results, including:

Income (*income*), needless to say, family income has a decisive influence on consumption. This article mainly uses the actual after-tax wages, bonuses, subsidy income and total income of industrial and commercial projects to characterize this indicator by all family members.

Fixed assets (*asset*), mainly based on the total asset value of industrial and commercial projects, the total value of self-owned vehicles (including cars, buses, electric bicycles, etc.) and the value of durable consumer goods (including refrigerators, televisions, washing machines, furniture, computers, etc.) are measured.

Cash holdings (*cash*), mainly include cash held by households, demand deposits, and time deposits.

Financial assets (*finance*), in modern economic society, financial products and services have a significant impact on household consumption. The indicators include stocks, funds, and bonds, gold, derivatives, wealth management products (including internet wealth management products, financial wealth management products), non-RMB assets, and other financial assets.

Commercial insurance (*security*), commercial insurance is mainly measured by the sum of the total insured amount of commercial health insurance and the total insured amount of commercial life insurance.

Debt (*debt*), most theoretical research believes that an appropriate amount of household debt will help increase consumption, and household debt is equivalent to borrowing from oneself in the future. From the perspective of alleviating current liquidity constraints, existing household debt has a positive effect on consumer spending effect. However, excessive debt usually leads to undesirable consequences [33]. Therefore, it is very necessary to introduce household debt as a control variable. This indicator covers bank credit (including industrial and commercial loans, housing loans, vehicle loans, and other non-financial asset loans, etc.), private loans.

Human capital (*human*), the stock of human capital is an important aspect that affects household income and consumption, because the education background and level of education of workers significantly affect output efficiency. The higher the level of human capital, the higher the rate of return to labor. The source of income and consumption expenditure provided to the family are also higher. This article combines the availability of data and uses the average years of education of family members to measure. Among them, the years of education at all levels are divided according to the following standards: 1 year without schooling, 6 years in primary school, 9 years in junior middle school, 12 years in senior high school and technical secondary school (vocational high school), 15 years in Junior College (Higher Vocational), 16 years in undergraduate course, 19 years in master’s degree and 22 years in doctoral degree.

The gender of the head of the household (*gender*), the main purpose of setting this variable is to control the bias caused by the family heterogeneity in the regression results, where the male is assigned a value of “1” and the female is assigned a value of “0”.

Family size (*scale*), family size is an important factor affecting consumption. Generally speaking, the larger the family population, the higher the total consumption of the family. So, we use the total number of family members to describe the family size.

Financial information (*information*), financial information is a key factor that affects household investment and consumption decision-making. Family or individuals will continue to understand relevant financial information and knowledge, which will help households to grasp the operating conditions of the market economy and reduce the cost of information collection and processing for investment decisions [34]. This variable mainly comes from the questionnaire “how much do you pay attention to economic and financial information” in the questionnaire and “what aspect of information do you primarily focus on”.

### 3.3. Data explanation and descriptive statistics

The empirical data used in this article is derived from the China Household Finance Survey and Research Center of Southwestern University of Finance and Economics in 2011, 2013, 2015, 2017, 2019, targeting 29 provinces (excluding Hong Kong, Macao, and Taiwan), 355 counties (districts, county-level cities), and 1428 communities across the country (village). The survey questionnaire covers household demographic characteristics, assets and liabilities, expenditure and income, insurance and security, financial knowledge, grass-roots governance and subjective attitude. It comprehensively reflects the family information of the respondents and is a typical large sample micro data. As the China Household Finance Survey (CHFS) project adopts a variety of measures to control sampling errors and non-sampling errors, the data quality is relatively high. In the specific data process, this article combines variable settings. First, the sample range is limited to urban households with housing, and the sample is further divided into three categories: first set of housing wealth appreciation, second set of housing wealth appreciation, and total housing wealth appreciation. After excluding outlier, seriously missing values and extreme values, the number of households corresponding to the three types of housing value-added samples is 50487, 13949 and 50487, respectively.

From the descriptive statistical results in **Table 2**, it can be found that in the observed sample of 50487 households, the average household consumption is 56005.7600 Yuan, and the average income is 56086.5600 Yuan. The first set of housing wealth appreciation, second set of housing wealth appreciation, and total housing wealth appreciation were 500989.8000 Yuan, 425451.5000 Yuan, and 926441.3000 Yuan respectively. The increase in property value is much higher than the increase in household income, and the appreciation of real estate is 16.52 times that of household income, indicating that household consumption may be affected by the increase in property value to a large extent. In addition, the averages of household financial assets and commercial insurance are 23920.3400 Yuan and 45048.7000 Yuan, respectively, indicating that the continuous improvement of China’s financial market system has significantly increased the proportion of household financial asset allocation.

**Table 2:** Descriptive statistics.

Name	Code	Unit	Mean	Min	Max	Sta. Dev.
Household consumption	*consumption*	Yuan	56005.7600	0.0000	1820000.0000	98412.8000
The first set of housing wealth appreciation	*house*_1_	Yuan	500989.8000	100.0000	10000000.0000	926883.7000
Second set of housing wealth appreciation	*house*_2_	Yuan	425451.5000	200.0000	8000000.0000	744679.1000
Total housing wealth appreciation	*house*_3_	Yuan	926441.3000	300.0000	18000000.0000	1109616.0000
Income	*income*	Yuan	56086.5600	0.0000	8000000.0000	299042.1000
Fixed assets	*asset*	Yuan	322987.6000	0.0000	8134000.0000	324000.0000
Cash holdings	*cash*	Yuan	25721.3400	0.0000	7000000.0000	112130.2000
Financial assets	*finance*	Yuan	23920.3400	0.0000	9850000.0000	355926.7000
Commercial insurance	*security*	Yuan	45048.7000	0.0000	9000000.0000	446156.0000
Debt	*debt*	Yuan	62595.2500	0.0000	7700000.0000	417475.0000
Human capital	*Human*	Year/person	8.0643	1.0000	22.0000	4.1495
The gender of the head of the household	*gender*	None	0.5019	0.0000	1.0000	0.4912
Family size	*scale*	Person	3.4004	1.0000	10.0000	1.5688
Financial information	*information*	None	0.4295	0.0000	1.0000	0.4950

## 4. Analysis of the impact of housing wealth appreciation on heterogeneous household consumption

### 4.1. Benchmark regression results

**Table 3** reports the estimation results of the benchmark model. Firstly, from the sample estimation results of the first set of housing wealth appreciation, at the 1% significance level, housing wealth appreciation has a positive effect on household consumption. The regression coefficient is 0.1293, which means that the housing wealth appreciation is 1%, and household consumption will increase significantly by 0.1293%. This means that the appreciation of house prices will help promote the consumption of urban households. Secondly, from the sample estimation results of the appreciation of the second set of houses, housing wealth appreciation has a positive effect on household consumption, the regression coefficient is 0.1197, and the coefficient is significant at the 1% significance level. This shows that if housing wealth appreciation increases by 1%, household consumption will increase significantly by 0.1197%. By comparing the results of column (1) and column (2), it is not difficult to find that the positive marginal contribution rate of the appreciation of the second set of houses to household consumption is greater than the appreciation of the first set of houses.

**Table 3:** Measurement results.

*lnconsumption*	(1)	(2)	(3)
*lnhouse*_1_	0.1293***		
	(35.00)		
*lnhouse*_2_		0.1197***	
		(11.77)	
*lnhouse*_3_			0.1354***
			(36.19)
*lnincome*	0.0264***	0.0216***	0.0255***
	(18.96)	(6.10)	(18.32)
*lnasset*	0.0475***	0.0563***	0.0461***
	(39.81)	(20.07)	(38.84)
*lncash*	0.0436***	0.0441***	0.0427***
	(21.05)	(8.04)	(20.66)
*lnfinance*	0.0181***	0.0174***	0.0172***
	(15.49)	(6.92)	(14.65)
*lnsecurity*	0.0184***	0.0144***	0.0179***
	(12.26)	(4.49)	(11.98)
*lndebt*	0.0248***	0.0198***	0.0244***
	(20.70)	(8.01)	(20.48)
*lnhuman*	0.1801***	0.2242***	0.1750***
	(19.75)	(8.25)	(19.13)
*gender*	0.0388***	0.0839***	0.0356***
	(3.62)	(3.06)	(3.33)
*lnscale*	0.3322***	0.3833***	0.3293***
	(29.87)	(11.75)	(29.65)
*information*	0.1681***	0.1387***	0.1652***
	(16.44)	(4.97)	(16.18)
*constant*	6.1980***	5.8749***	5.9820***
	(113.61)	(39.72)	(103.29)
*dum*_ *household*	Yes	Yes	Yes
*dum*_*year*	Yes	Yes	Yes
*dum*_*province*	Yes	Yes	Yes
*R* ^2^	0.46	0.55	0.47
*F*-*statistics*	460.27***	139.51***	456.31***
*N*	50487	13949	50487

**Notes:** ***, ** and * are significant at the significance level of 1%, 5%, and 10%, respectively. The number in parentheses below the regression coefficient is the robust standard error t value, the same below.

Further analysis of the sample estimation results of the total value of real estate, the value of real estate has a positive effect on household consumption, the regression coefficient is 0.1354, and this coefficient is significant at the 1% significance level. This shows that if housing wealth appreciation increases by 1%, household consumption will increase significantly by 0.1354%. Finally, observe the estimated results of the control variables. On the whole, income, fixed assets, cash holdings, financial assets, commercial insurance, debt, human capital, family size, financial information have a positive effect on household consumption. By the way, the regression coefficients of income, fixed assets, cash holdings and other variables are significant at the significance level of 1% respectively, which is in line with the expectations of this article. It can be seen that under the control of economic characteristics, individual characteristics, household characteristics, etc., housing wealth appreciation has a significant positive effect on household consumption. The results are also consistent with the basic conclusions of relevant studies, such as Bostic et al. (2009) [35].

### 4.2. Heterogeneity regression results

Whether a family can obtain a real estate mortgage loan from a commercial bank is an important manifestation of the role of credit constraints as a guarantee. Since this article emphasizes heterogeneous households, it is necessary to analyze the impact of credit constraints on household consumption. In fact, it is not easy to tell whether a family is subject to credit constraints. The foregoing theoretical mechanism shows that household consumption is significantly affected by credit constraints, and housing wealth appreciation can ease household borrowing constraints and increase consumer spending through refinancing. However, for savings households that are subject to strong credit constraints, they are more sensitive to savings and less willing to increase household consumption. To this end, this article draws on the ideas of Zeldes (1989), Jappelli et al. (1998), and Gan (2010) [36–38], from the number of real estate ownership, housing loan application status, and credit card holding status, according to the questionnaire “How many houses your family has in total”, “Is there a bank mortgage loan” and “Does your family use a credit card”. Three choices to measure whether the household faces credit constraints. If the option of how many houses a household owns is “1”, the “household credit constraint” is equal to 1, otherwise it is 0. If the reason why the family doesn’t choose to buy a house is “no money to pay the down payment or no mortgage repayment ability”, the “household credit constraint” is equal to 1, otherwise it is 0. If the option of whether the family uses a credit card (inactive credit cards are not included) is “no”, the “family credit constraint” is equal to 1, otherwise it is 0. **Table 4** lists the econometric results of household consumption under different credit constraints. The analysis is as follows:

**Table 4:** Measurement results.

*lnconsumption*	(4)	(5)	(6)	(7)	(8)	(9)
	A house	Multiple houses	Loan restricted	Non-loan restricted	No credit card	Have a credit card
*lnhouse*_1_	0.1291***	0.1293***	-0.1490***	0.1211***	0.1097***	0.1286***
	(31.22)	(12.62)	(-6.98)	(27.07)	(7.33)	(29.84)
*lnincome*	0.0269***	0.0237***	0.0200***	0.0251***	0.0264***	0.0247***
	(14.62)	(6.47)	(4.47)	(15.05)	(6.02)	(14.12)
*lnasset*	0.0405***	0.0587***	0.0689***	0.0450***	0.0499***	0.0444***
	(27.85)	(18.35)	(13.85)	(34.45)	(12.72)	(29.83)
*lncash*	0.0389***	0.0492***	0.0483***	0.0466***	0.0366***	0.0457***
	(17.06)	(8.14)	(7.54)	(15.70)	(5.71)	(19.24)
*lnfinance*	0.0148***	0.0163***	0.0109**	0.0160***	0.0105***	0.0197***
	(9.29)	(5.80)	(2.43)	(10.86)	(3.69)	(11.72)
*lnsecurity*	0.0126***	0.0240***	0.0295***	0.0109***	0.0153***	0.0179***
	(6.80)	(6.77)	(5.90)	(5.24)	(4.63)	(8.65)
*lndebt*	0.0251***	0.0235***	0.0239***	0.0253***	0.0172***	0.0250***
	(16.16)	(8.40)	(4.68)	(17.44)	(5.19)	(15.51)
*lnhuman*	0.1687***	0.2651***	0.3651***	0.1412***	0.1360***	0.1630***
	(15.56)	(9.30)	(8.12)	(13.79)	(4.45)	(14.82)
*gender*	0.0314**	0.0426	-0.0603	0.0282**	0.0556**	0.0445***
	(2.42)	(1.52)	(-1.60)	(2.18)	(1.98)	(3.24)
*lnscale*	0.3525***	0.3223***	0.4985***	0.2577***	0.1746***	0.3612***
	(26.84)	(10.11)	(10.97)	(19.16)	(4.91)	(26.89)
*information*	0.1717***	0.1460***	0.1813***	0.1702***	0.0881***	0.1628***
	(13.91)	(4.97)	(4.69)	(13.37)	(2.68)	(12.93)
*constant*	6.3323***	5.9147***	5.0593***	6.4062***	7.3500***	6.1912***
	(83.47)	(39.82)	(22.40)	(77.92)	(43.31)	(96.74)
*dum*_ *household*	Yes	Yes	Yes	Yes	Yes	Yes
*dum*_*year*	Yes	Yes	Yes	Yes	Yes	Yes
*dum*_*province*	Yes	Yes	Yes	Yes	Yes	Yes
*R* ^2^	0.39	0.57	0.59	0.41	0.39	0.43
*F*-*statistics*	230.98***	150.35***	98.16***	288.61***	41.39***	267.03***
*N*	34283	16204	15542	34945	11034	30453

First, from the results of the number of real estate ownership, the results of column (4) show that at a significance level of 1%, housing wealth appreciation has a positive effect on household consumption, and the regression coefficient is 0.1291, which means that housing price appreciation is 1%, Then household consumption will increase significantly by 0.1291%. The results of column (5) show that at a significance level of 1%, housing wealth appreciation has a positive effect on household consumption, and the regression coefficient is 0.1293, which means that if the house price increases by 1%, household consumption will increase significantly by 0.1293%. By comparing the results of column (4) and column (5), it is not difficult to find that the positive marginal contribution rate of multiple houses to household consumption is greater than that of one house. It can be seen that for families with multiple houses, that is, families with less credit constraints, the marginal contribution rate of property appreciation to household consumption is greater, and vice versa.

Second, from the results of the housing loan application status, the results of column (6) show that at a significance level of 10%, housing wealth appreciation has a negative effect on household consumption, and the regression coefficient is -0.1490, indicating that if the housing price increases by 1%, household consumption will significantly decrease by 0.1490%. The results of column (7) show that at the significance level of 1%, housing wealth appreciation has a positive effect on household consumption, and the regression coefficient is 0.1211, indicating that if house price increases by 1%, household consumption will increase significantly by 0.1211%. By comparing the results of column (6) and column (7), it is not difficult to find: On the one hand, for households with limited loans, that is, households with greater credit constraints, housing wealth appreciation will produce “crowding-out effect” on household consumption. On the other hand, for non-loan restricted households, that is, households with less credit constraint, housing wealth appreciation is conducive to promoting household consumption expenditure.

Third, from the results of credit card holdings, the results of column (8) indicate that at a significance level of 1%, the appreciation of housing wealth has a positive impact on household consumption, with a regression coefficient of 0.1097, indicating that if housing prices rise by 1%, household consumption will significantly increase by 0.1097%. The results of column (9) indicate that, at a significance level of 1%, the appreciation of housing wealth has a positive impact on household consumption, with a regression coefficient of 0.1286. On the one hand, for households without credit cards, i.e. households with greater credit constraints, the marginal contribution of housing wealth appreciation to household consumption is smaller. On the other hand, for households with credit cards, i.e. those with less credit constraints, the appreciation of housing wealth is beneficial for promoting household consumption expenditures.

The above results show that the effect of housing wealth appreciation on household consumption under different credit constraints is heterogeneous. Among them, housing wealth appreciation has a significant positive impact on the consumption expenditure of households with multiple houses, non-restricted loans, and credit cards. It is not difficult to understand that for a type of households with weaker credit constraints (borrowing households), they tend to obtain financing through real estate mortgage loans, so that they can invest in more real estate, and use housing wealth appreciation to achieve more wealth income. In the process of rising housing prices, it will undoubtedly help promote household consumption expenditure. On the contrary, for the savings households that are subject to strong credit constraints, because it is difficult to obtain commercial bank mortgages, rising house prices will only further increase the size of household savings and reduce household consumption expenditures, that is, savings households are more liquid.

### 4.3. Quantile regression results

Next, in order to examine in detail the marginal propensity to consume of household consumption by housing wealth appreciation, this paper presents the measurement and estimation results of the five quantiles (q_10, q_30, q_50, q_70 and q_90), see the **Tables 5–7** and **Fig 2**, the following analysis in turn:

**Table 5:** Measurement results.

*lnconsumption*	(10)	(11)	(12)	(13)	(14)
	q_10	q_30	q_50	q_70	q_90
*lnhouse*_1_	0.1201***	0.1305***	0.1423***	0.0980***	0.0985***
	(29.71)	(40.34)	(31.19)	(24.02)	(17.58)
*lnincome*	0.1067***	0.0579***	0.0366***	0.0281***	0.0270***
	(23.87)	(29.27)	(29.19)	(21.27)	(12.48)
*lnasset*	0.0326***	0.0321***	0.0377***	0.0396***	0.0433***
	(12.01)	(19.41)	(27.13)	(30.44)	(19.27)
*lncash*	0.0388***	0.0248***	0.0180***	0.0139***	0.0089***
	(8.43)	(9.80)	(9.39)	(6.82)	(2.77)
*lnfinance*	-0.0093***	0.0106***	0.0206***	0.0300***	0.0484***
	(-3.14)	(5.54)	(11.85)	(18.56)	(14.46)
*lnsecurity*	0.0039	0.0029	0.0073***	0.0140***	0.0308***
	(1.13)	(1.43)	(3.88)	(7.04)	(8.38)
*lndebt*	0.0020	0.0092***	0.0109***	0.0156***	0.0234***
	(0.65)	(5.55)	(8.68)	(11.52)	(8.41)
*lnhuman*	0.2667***	0.2832***	0.2121***	0.1659***	0.1285***
	(10.53)	(19.41)	(20.38)	(15.93)	(8.48)
*gender*	-0.3037***	-0.1353***	-0.0465***	-0.0165	0.0081
	(-10.55)	(-9.38)	(-4.47)	(-1.55)	(0.43)
*lnscale*	0.3970***	0.4240***	0.3437***	0.2971***	0.3395***
	(10.96)	(27.59)	(29.66)	(25.06)	(15.36)
*information*	0.1192***	0.0861***	0.0469***	0.0473***	0.0497***
	(5.65)	(5.93)	(4.75)	(3.79)	(2.47)
*constant*	3.1581***	5.5097***	7.3461***	8.4177***	9.0371***
	(15.95)	(58.86)	(104.08)	(132.63)	(98.57)
*dum*_ *household*	Yes	Yes	Yes	Yes	Yes
*dum*_*year*	Yes	Yes	Yes	Yes	Yes
*dum*_*province*	Yes	Yes	Yes	Yes	Yes
*R* ^2^	0.21	0.30	0.30	0.25	0.14
*N*	50487	50487	50487	50487	50487

**Table 6:** Measurement results.

*lnconsumption*	(15)	(16)	(17)	(18)	(19)
	q_10	q_30	q_50	q_70	q_90
*lnhouse*_2_	0.0976***	0.1086***	0.1361***	0.1077***	0.1164***
	(4.02)	(6.00)	(9.20)	(8.33)	(6.36)
*lnincome*	0.0920***	0.1281***	0.0938***	0.0533***	0.0323***
	(12.01)	(21.49)	(16.98)	(12.67)	(5.80)
*lnasset*	0.0553***	0.0675***	0.0725***	0.0726***	0.0640***
	(7.20)	(12.18)	(15.79)	(19.92)	(12.26)
*lncash*	0.0365***	0.0001	-0.0055	0.0121**	-0.0026
	(2.97)	(0.01)	(-0.86)	(2.15)	(-0.31)
*lnfinance*	-0.0076	0.0094*	0.0233***	0.0334***	0.0393***
	(-1.11)	(1.89)	(6.78)	(8.03)	(5.42)
*lnsecurity*	0.0029	-0.0017	-0.0001	0.0090**	0.0292***
	(0.40)	(-0.27)	(-0.03)	(2.06)	(3.66)
*lndebt*	0.0076	0.0109**	0.0130***	0.0136***	0.0174***
	(1.24)	(2.22)	(3.92)	(3.93)	(2.97)
*lnhuman*	0.1426***	0.0391	0.0754**	0.1140***	0.1020***
	(2.74)	(0.78)	(2.37)	(3.59)	(2.60)
*gender*	-0.1448**	-0.1978***	-0.0779**	-0.0425	0.0301
	(-2.36)	(-4.88)	(-2.28)	(-1.37)	(0.63)
*lnscale*	0.4919***	0.2284***	0.2586***	0.2720***	0.3222***
	(5.76)	(4.28)	(6.01)	(6.51)	(5.75)
*information*	0.2400***	0.2047***	0.1132***	0.0331	0.1070**
	(3.98)	(4.56)	(2.88)	(0.98)	(2.13)
*constant*	4.7451***	6.2240***	6.8464***	7.9462***	8.9120***
	(11.92)	(23.58)	(33.05)	(43.34)	(34.77)
*dum*_ *household*	Yes	Yes	Yes	Yes	Yes
*dum*_*year*	Yes	Yes	Yes	Yes	Yes
*dum*_*province*	Yes	Yes	Yes	Yes	Yes
*R* ^2^	0.17	0.39	0.44	0.34	0.17
*N*	13949	13949	13949	13949	13949

**Table 7:** Measurement results.

*lnconsumption*	(20)	(21)	(22)	(23)	(24)
	*q*_10	*q*_30	*q*_50	*q*_70	*q*_90
*lnhouse*_3_	0.1207***	0.1424***	0.1249***	0.0920***	0.0991***
	(22.03)	(36.01)	(35.31)	(23.34)	(14.19)
*lnincome*	0.1225***	0.0681***	0.0428***	0.0323***	0.0311***
	(27.02)	(31.11)	(29.70)	(23.56)	(15.55)
*lnasset*	0.0377***	0.0346***	0.0388***	0.0401***	0.0434***
	(12.41)	(19.86)	(29.55)	(30.35)	(20.93)
*lncash*	0.0333***	0.0208***	0.0154***	0.0119***	0.0067**
	(7.17)	(8.01)	(8.14)	(5.44)	(2.35)
*lnfinance*	-0.0065**	0.0116***	0.0209***	0.0299***	0.0479***
	(-2.19)	(6.16)	(13.77)	(18.09)	(13.47)
*lnsecurity*	0.0028	0.0020	0.0068***	0.0135***	0.0303***
	(0.76)	(1.06)	(3.32)	(7.10)	(8.09)
*lndebt*	-0.0028	0.0061***	0.0090***	0.0143***	0.0220***
	(-0.96)	(3.87)	(6.33)	(9.02)	(9.77)
*lnhuman*	0.2967***	0.2953***	0.2165***	0.1657***	0.1248***
	(11.24)	(18.95)	(20.46)	(16.74)	(8.27)
*gender*	-0.3764***	-0.1836***	-0.0769***	-0.0373***	-0.0130
	(-13.65)	(-13.65)	(-6.52)	(-3.63)	(-0.79)
*lnscale*	0.3576***	0.3989***	0.3286***	0.2870***	0.3296***
	(14.07)	(23.59)	(24.86)	(22.77)	(14.82)
*information*	0.1009***	0.0726***	0.0379***	0.0406***	0.0423***
	(3.59)	(4.80)	(3.80)	(3.66)	(2.12)
*constant*	3.9672***	5.9023***	7.5300***	8.4869***	9.0413***
	(23.65)	(60.42)	(130.04)	(154.44)	(83.84)
*dum*_ *household*	Yes	Yes	Yes	Yes	Yes
*dum*_*year*	Yes	Yes	Yes	Yes	Yes
*dum*_*province*	Yes	Yes	Yes	Yes	Yes
*R* ^2^	0.19	0.28	0.29	0.25	0.14
*N*	50487	50487	50487	50487	50487

First of all, from the quantile measurement of the first set of housing wealth appreciation, when the consumption level is located at the q_10 and q_30, the housing wealth appreciation has a positive impact on the household consumption, and the marginal effect coefficients are 0.1201 and 0.1305 respectively, and both are significant at the significance level of 1%. When the household consumption level is located at q_50, the marginal effect coefficient is 0.1423, which is significant at the significance level of 1%. When the household consumption level is located at the q_70 and q_90, the housing wealth appreciation has a positive impact on the household consumption, and the marginal effect coefficients of are 0.0980 and 0.0985 respectively, and both are significant at the significance level of 1%. In more detail, from the evolutionary trend chart of the marginal effect of housing wealth appreciation on household consumption, the marginal effect coefficient of housing wealth appreciation on household consumption shows a gradual increasing trend from q_10 to q_50, and reaches the peak at q_50. After q_50, there was a downward trend, but the marginal effect coefficient remained positive. Overall, housing wealth appreciation has a positive role in promoting household consumption, its marginal effect coefficient shows a trend of increasing first and then decreasing, which means influence of housing wealth appreciation of the marginal effect of household consumption has a inverted “U” type evolution.

Secondly, from the quantile measurement of the second set of housing wealth appreciation, when the household consumption is at the q_10, q_30 and q_50, the housing wealth appreciation has a positive impact on the household consumption, and the marginal effect coefficients are 0.0976, 0.1086 and 0.1361, respectively. Therefore, for the sample of the wealth appreciation of the family with two houses, when the household consumption is in the conditional distribution from q_10 to q_50, as the household consumption level increases, the degree of influence of the housing wealth appreciation on it will gradually increase. Furthermore, when household consumption is located at the q_70 and q_90 quintiles, housing wealth appreciation has a positive impact on household consumption. The marginal effect coefficients are 0.1077 and 0.1164, respectively, and they are significant at the significance levels of 1%. Compared with the q_50 points, the coefficient of marginal effect of housing wealth appreciation on household consumption has been weakened. From the perspective of the evolution trend chart of the marginal effect of housing wealth appreciation affecting household consumption, within the range of q_10 to q_30, the marginal effect coefficient of housing wealth appreciation on household consumption shows a gradual upward trend. However, after the q_30 quintile, its marginal effect coefficient showed a gradual decline trend, and reached a trough at the q_70 quintile, and showed an upward trend after the q_70 quintile. From the sample of the second set of housing wealth appreciation, the impact of the appreciation of real estate on household consumption will show complex changes according to the level of household consumption.

Finally, from the quantile measurement results of the total housing wealth appreciation, when the household consumption level is at the q_10 and q_30, the housing wealth appreciation has a significant positive impact on household consumption, and the marginal effect coefficients of the two are 0.1207 and 0.1424, respectively, and the marginal effect coefficient is greater than 0.1000. When the household consumption level is located at the q_50, q_70 and q_90, housing wealth appreciation has a positive impact on household consumption, and its marginal effect coefficients are 0.1249, 0.0920 and 0.0991 respectively, and are significant at the 1% significance level. Therefore, for the sample of total housing wealth appreciation, when household consumption is in the conditional distribution from q_50 to q_90, as household consumption levels increase, the degree of housing wealth appreciation on its influence is a basic trend of decline. Furthermore, from the perspective of the evolution trend chart of the marginal effect of housing wealth appreciation on household consumption, in the range of q_10 to q_50, the coefficient of marginal effect of housing wealth appreciation on household consumption shows a monotonically increasing trend. After the q_50, its marginal effect coefficient showed a gradual downward trend, but the value of the marginal effect coefficient is less than 0.1000. In general, the total housing wealth appreciation has a relatively even impact on the distribution of household consumption conditions. With the increase in household consumption levels, it mainly presents a basic trend of gradual increase in household consumption.

This article concludes that the “wealth amplification effect” produced by housing wealth appreciation will stimulate household consumption on the whole. For every 1% of housing wealth appreciation, household consumption will significantly increase by about 0.10–0.14%. In the past, the impact of house prices or real estate on household consumption was mostly analyzed based on the “wealth effect”, which means that the rise of house prices means the increase of wealth for those who have houses, so it can better promote household consumption. The “wealth amplification effect” proposed in this paper is integrated into the “mortgage effect” of real estate, that is, homeowners obtain loans through mortgaged real estate to obtain more funds. The looser the degree of credit constraints, the rise of house prices will further alleviate the degree of credit constraints of house buyers, so as to provide more support for family consumption. Compared with the “wealth effect”, the “wealth amplification effect” includes not only the increase of wealth caused by the rise of house prices, but also the funds obtained by means of real estate mortgage. It is the mechanism and process of the interaction between “wealth effect” and “mortgage effect”. Therefore, from the perspective of expanding domestic demand, we should give full play to the decisive role of the real estate market in the process of housing allocation, and constrain real estate by optimizing the allocation of credit resources investment behavior, thereby reducing income inequality and promoting household consumption.

## 5. Robustness test and further analysis

In this study, there may be endogenous problems due to missing variables or reverse causality. On the one hand, the influencing factors of household consumption are complex. In addition to some family characteristics and head of household characteristic variables controlled in this paper, household consumption is also affected by personal habits, personality, social atmosphere, information acquisition and other factors, which are difficult to observe and quantify. On the other hand, there may be a causal correlation between housing wealth and household consumption, housing as a commodity, the higher the level of household consumption, the higher the house price that can be paid, and the higher the consumption level of the region indicates the higher the degree of economic development, the higher the possibility of housing wealth appreciation. Therefore, this paper needs to introduce tool variables to analyze the robustness of the empirical conclusions. This article mainly selects housing property right and land transfer as tool variables. In this article, the property rights of real estate are set based on the ‘property rights form of this house’ in the questionnaire. Among them, all property rights are ‘1’. Partial property rights, minor property rights, and others have a value of ‘0’. Land transfer refers to the transfer area of residential land in the province where the family real estate is located (unit: Hectare). The specific reasons are as follows: Firstly, different forms of property rights result in significant differences in housing prices. If households do not own all property rights, are unable to buy or sell, or have strict buying and selling conditions and poor liquidity, this will undoubtedly affect the appreciation of property. At the same time, there is no inevitable connection between property rights and household consumption levels. Therefore, housing property rights is a reasonable tool variable for housing wealth appreciation. Second, the tightening of land supply in China will directly lead to a rapid rise in housing prices, because the scarcity of land will make land prices rise, thus leading to a rise in urban housing prices. However, the amount of land supply will not affect household consumption, and there is no obvious correlation between them. Therefore, land transfer is a reasonable tool variable of housing price. Results of robustness based 2SLS are reported in Table 8, analyzed in order below.

**Table 8:** Robustness test results.

	(25)	(26)	(27)	(28)
	*lnhouse*_1_	*lnconsumption*	*lnhouse*_2_	*lnconsumption*
*lnhouse*_1_		0.1159**		
		(2.19)		
*lnhouse*_2_				0.8986**
				(2.29)
*lnincome*	0.0600***	0.1653***	0.0278***	0.1389***
	(32.54)	(3.89)	(10.32)	(6.85)
*lnasset*	0.0593***	0.1495***	0.0506***	0.1301***
	(37.56)	(3.50)	(20.11)	(3.66)
*lncash*	0.0153***	0.0625***	0.0480***	0.1081***
	(4.78)	(4.83)	(9.34)	(3.08)
*lnfinance*	0.0491***	0.1004***	0.0464***	0.0746**
	(25.46)	(2.85)	(16.72)	(2.29)
*lnsecurity*	-0.0021	0.0022	0.0149***	0.0332***
	(-0.74)	(0.40)	(4.01)	(2.83)
*lndebt*	-0.0156***	-0.0194*	-0.0062**	0.0066
	(-8.28)	(-1.69)	(-2.28)	(1.22)
*lnhuman*	0.2629***	0.6311***	0.3059***	0.5765***
	(23.04)	(3.35)	(13.89)	(2.70)
*gender*	-0.1319***	-0.2960***	0.0182	-0.1695***
	(-8.68)	(-2.96)	(0.80)	(-4.46)
*lnscale*	-0.2552***	-0.2378	-0.1575***	0.2505**
	(-14.54)	(-1.27)	(-5.20)	(1.96)
*information*	0.0936***	0.2268***	0.0666**	0.2149***
	(5.85)	(3.13)	(2.60)	(3.49)
*right*	0.0391**		0.0262**	
	(2.38)		(2.19)	
*lnland*	-0.0154*		-0.0312*	
	(-1.76)		(-1.69)	
*constant*	10.4215***	24.6380***	9.8963***	16.5032**
	(104.98)	(3.35)	(52.47)	(2.32)
*dum*_ *household*	Yes	Yes	Yes	Yes
*dum*_*year*	Yes	Yes	Yes	Yes
*dum*_*province*	Yes	Yes	Yes	Yes
*R* ^2^	0.29	0.34	0.37	0.44
*F*-*statistics*	420.35***		198.36***	
*Wald-value*		1872.62***		3560.67***
*N*	37823	37823	11859	11859

**Notes:** We are unable to obtain land transfer data for each city in 2019, so the results in the table mainly use the CHFS data in 2011, 2013, 2015 and 2017.

In **Table 8**, the results of first stage model of the first set of housing wealth appreciation sample shows that: Firstly, the housing property right has a positive impact on the value of the house, with the regression coefficient of 0.0391, which is in line with the expectations of this article. At the same time, land transfer has a negative impact on housing wealth appreciation, and the regression coefficient is -0.0154, indicating that the larger the land supply area, the more conducive to inhibiting the rise of housing price, which meets the expectations of this article. Second, the econometric results of the 2SLS stage two column of the whole sample, model (26) showed that housing wealth appreciation promoted household consumption with a regression coefficient of 0.1159, which is consistent with the previous conclusion.

Next, this paper further analyzes the results of first stage model of the second set of housing wealth appreciation sample: Firstly, the housing property right has a positive impact on housing wealth appreciation, with a regression coefficient of 0.0262, which is consistent with the above. At the same time, land transfer had a negative effect on housing wealth appreciation, with a regression coefficient of -0.0312 and was significant at the 10% significance level, which is expected in this paper. Secondly, results of full sample of 2SLS stage two of the column (28) showed that housing wealth appreciation promoted household consumption with a regression coefficient of 0.8986. Compared with the previous results, the robustness test did not change the direction of the role of housing wealth appreciation in influencing household consumption, which shows that the research conclusion of this paper is robustness.

## 6. Conclusions and policy implications

By introducing the financial friction of the credit mortgage constraint mechanism, this paper reveals the smoothing effect of real estate mortgage on the current and future consumption path, and empirically tests the theoretical mechanism by using the CHFS data in 2011, 2013, 2015, 2017, and 2019 which provides a new explanation for the degree of liquidity constraints and consumption differences faced by heterogeneous families. Through the research, the conclusions and policy implications of this paper are as follows:

Firstly, the financial accelerator mechanism plays an important role in the transmission process of house price impact to household consumption. Real estate mortgage helps to alleviate the plight of insufficient household liquidity. Borrowing households tend to obtain loans through mortgage real estate to smooth the consumption of each period. The looser the credit constraint, the more obvious the rise of house price on the consumption expenditure of borrowing households, but the weaker the consumer spending on savings households. It can be seen that expanding domestic demand distinguishes different types of households, and takes seriously the impact of real estate mortgage loans on household consumption. Only by further standardizing and deepening the real estate financing market can the benefits brought by housing wealth appreciation be transformed into effective consumption capacity.

Secondly, the impact of housing wealth appreciation on household consumption under different credit constraints is heterogeneous. Among them, housing wealth appreciation has a significant positive impact on household consumption expenditure with multiple houses, credit cards, non-loan restrictions, while the marginal effect on the consumption expenditure of households with only one house, loan limited, and no credit cards decreases. Therefore, we should improve the credit services in the field of first house and indemnificatory housing, implement the differentiated housing credit supply strategy, increase the credit demand for rigid house purchase, curb the credit demand for speculative house purchase, and avoid excessive investment in the real estate market. While meeting the normal housing demand of families, so as to optimize the loan supply to achieve the balance between supply and demand in the real estate market.

Thirdly, on the whole, the “wealth amplification effect” generated by housing wealth appreciation will stimulate household consumption. For every 1% housing wealth appreciation, household consumption will increase significantly by about 0.10–0.14%. At the same time, we also discusses the endogeneity of the core explanatory variables, and uses 2SLS to effectively verify the robustness of the conclusions. This means that we should dialectically look at the economic and social problems caused by the rise of house prices. While abandoning real estate as an economic stimulus, improving the efficiency of urban and rural land resource allocation to reduce urban house prices, optimizing urban scale and stimulating national consumption will be the key issue to promote the reform of the real estate market in the future.

Accordingly, this paper puts forward the following policy recommendations: Firstly, support the in-depth development of the real estate market, promote the development of the real estate Financial innovation and market, optimize the allocation of real estate assets of Chinese households, improve the real income level of residents through the optimal allocation of real estate resources, and enhance the purchasing power of families, which is the driving force to promote consumption. Secondly, to improve the real estate market and related credit markets, housing mortgage loans can relax the constraints on households purchases. To this end, for families with insufficient liquidity assets, fully leverage the dynamic role of the real estate mortgage market, so that housing can alleviate the liquidity constraints faced by families and promote household consumption. Thirdly, on the basis of strengthening macro prudential supervision, we should stabilize the real estate price. For single housing families with insufficient liquidity assets, on the one hand, we should ensure that their housing assets will not shrink and their consumption capacity will be stabilized. On the other hand, we should increase the income of these families, so that residents can better share the wealth effect brought by the appreciation of real estate, and then stimulate residents’ consumer behaviour. At the same time, it is necessary to control the level of housing debt as much as possible, reduce the burden of housing debt, promote the accumulation of household liquidity assets, and thus achieve better release of household consumption potential.
